# Death from Inoculation with Matter from an Ulcer Succeeding the Vaccine Vesicle

**Published:** 1846-04

**Authors:** Daniel Mowe

**Affiliations:** Lowell


16. *Death from inoculation with matter from an ulcer succeeding the vaccine vesicle.*—On the fourteenth day from vaccination, when there was no vaccine matter present, but a foul secretion, this fluid was taken by needles, and inserted into the arms of two children by their mother or some one present. The operation was performed on the 29th of Sept. at 8½ o'clock, A. M. At 8 o'clock P. M., both were taken ill suddenly, with much restlessness and great heat, the same symptoms in both. At 10 o'clock P. M., symptoms somewhat worse; but the parents thinking they would soon be better, no medical aid was called until 2 o'clock at night, fourteen hours after vaccination, as they termed it, when the infant, 18 months old, became convulsed. Dr. Allen, of this city, being near, was called in. Spasms continuing, at 8 o'clock, Sept. 30th, I met Dr. A., and we continued in attendance till 10½ o'clock, when it expired, twenty-six hours after inoculation. The virus corroded through the true skin in this short period, and arm swollen.

The other child, about four years old, continued very sick; arm swollen, red and painful, with a white ulcer, evidently extended through the true skin. There was no vaccine vesicle or scab, or anything like it, formed on the arm. The ulcer continued to discharge until about the 10th of January, 1846; and on the 19th of January, the last scab fell off, leaving an unsound appearance, and a hardness in the cellular membrane beneath. It is evident that a virulent poison was introduced into the arms of these children. DANIEL MOWE.

*Lowell, Feb.* 5, 1846. [*Boston Med. and Surg. Jour.*

